# Amplitude of Sensorimotor Mu Rhythm Is Correlated with BOLD from Multiple Brain Regions: A Simultaneous EEG-fMRI Study

**DOI:** 10.3389/fnhum.2016.00364

**Published:** 2016-07-22

**Authors:** Siyang Yin, Yuelu Liu, Mingzhou Ding

**Affiliations:** ^1^J. Crayton Pruitt Family Department of Biomedical Engineering, University of Florida, GainesvilleFL, USA; ^2^Center for Mind and Brain, University of California, Davis, DavisCA, USA

**Keywords:** simultaneous EEG-fMRI, mu rhythm, SOBI, resting-state, motor control, attention control, mirror neurons, salience network

## Abstract

The mu rhythm is a field oscillation in the ∼10Hz range over the sensorimotor cortex. For decades, the suppression of mu (event-related desynchronization) has been used to index movement planning, execution, and imagery. Recent work reports that non-motor processes, such as spatial attention and movement observation, also desynchronize mu, raising the possibility that the mu rhythm is associated with the activity of multiple brain regions and systems. In this study, we tested this hypothesis by recording simultaneous resting-state EEG-fMRI from healthy subjects. Independent component analysis (ICA) was applied to extract the mu components. The amplitude (power) fluctuations of mu were estimated as a time series using a moving-window approach, which, after convolving with a canonical hemodynamic response function (HRF), was correlated with blood-oxygen-level-dependent (BOLD) signals from the entire brain. Two main results were found. First, mu power was negatively correlated with BOLD from areas of the sensorimotor network, the attention control network, the putative mirror neuron system, and the network thought to support theory of mind. Second, mu power was positively correlated with BOLD from areas of the salience network, including anterior cingulate cortex and anterior insula. These results are consistent with the hypothesis that sensorimotor mu rhythm is associated with multiple brain regions and systems. They also suggest that caution should be exercised when attempting to interpret mu modulation in terms of a single brain network.

## Introduction

The mu rhythm (8 to 12 Hz) is a field oscillation observed over central and central-parietal electrodes in humans. Since its discovery, the suppression or blocking of mu (desynchronization) has been associated with a variety of movement conditions, including passive movements, reflexive movements, voluntary movements, and commanded movements, as well as with tactile stimulation (Chatrian et al., 1959). Subsequent work demonstrates that motor planning or even motor imagery can desynchronize mu (Pfurtscheller and Neuper, 1997). The sensorimotor origin of mu modulation was further solidified in simultaneous EEG-fMRI studies where the power of mu was found to be negatively correlated with blood-oxygen-level-dependent (BOLD) signals from the pericentral cortex and supplementary motor cortex (Ritter et al., 2009; Mizuhara, 2012).

Association of mu with other brain regions and neural systems has also been suggested. First, observation of movements can suppress mu power (Cohen-Seat et al., 1954; Cochin et al., 1999; Pineda, 2005), suggesting a link between mu and the mirror neuron system (Pineda, 2005). In humans, areas implicated in mirror neuron activity include premotor cortex, considered as the homolog to area F5 of the macaque monkey where the mirror neurons were initially discovered (Rizzolatti et al., 1996; Buccino et al., 2001; Keysers and Gazzola, 2009), inferior parietal lobule (IPL) (Fogassi et al., 2005), inferior frontal gyrus (IFG) (Kilner et al., 2009), and posterior middle temporal gyrus (pMTG) (Gazzola et al., 2007). It is thus reasonable to expect that mu is associated with the activity of these brain regions by exhibiting negative correlation with their BOLD.

Second, theory of mind, a hypothesized mechanism to comprehend the mental states of others, can be thought of as an extension of the mirror neuron concept to the cognitive and affective domains. Brain regions that are frequently implicated in theory of mind include medial prefrontal cortex (mPFC), superior temporal sulcus/ superior temporal gyrus (STS/STG), temporoparietal junction (TPJ), and temporal poles (TP) (Schulte-Rüther et al., 2007; Mar, 2011). Although no prior studies have reported a relationship between these regions and the mu rhythm, in light of the resemblance between the mirror neuron mechanism and theory of mind, it is reasonable to expect that the BOLD signal from theory of mind regions and the power of mu may be inversely correlated.

Third, mu rhythm has been shown to be somatotopically modulated by spatial somatosensory attention (Jones et al., 2010; Anderson and Ding, 2011), analogous to the modulation of visual alpha rhythm by spatial visual attention (Vanni et al., 1997; Worden et al., 2000; Capotosto et al., 2009; Rajagovindan and Ding, 2011). This raises the possibility that attention control regions including intraparietal sulcus (IPS) and frontal eye field (FEF) are inversely coupled with the mu rhythm.

In this paper we tested these hypotheses by recording simultaneous EEG-fMRI from healthy subjects at rest. Mu components were isolated using the independent component analysis (ICA) method. The power of mu fluctuations was extracted from these ICA components using short-time Fourier transforms and convolved with a canonical hemodynamic response function (HRF). The HRF-convolved mu power time series were then correlated with the concurrently recorded BOLD activity to assess both negative and positive couplings between mu and neural activity in different brain regions.

## Materials and Methods

### Experimental Procedure

The experimental protocol and data acquisition procedure were approved by the Institutional Review Board of the University of Florida. Thirty-six healthy, right-handed college students (19 females; mean age: 20.45 ± 3.66) gave informed consent and participated in the study in exchange for course credits. During resting-state recording (7 min) subjects were instructed to lie still in the scanner with eyes closed and refrain from falling asleep or focusing on any specific thoughts.

### EEG Data Acquisition

EEG data were recorded using a 32-channel MR-compatible EEG system (Brain Products GmbH, Germany). Thirty one sintered Ag/AgCl electrodes were placed according to the 10–20 system and one additional electrode was placed on the participant’s upper back to monitor electrocardiogram (ECG). Electrocardiogram was used subsequently to aid the removal of the cardioballistic artifacts. The impedance from all scalp channels was kept below 10 kΩ during the entire recording session as recommended by the manufacturer.

The online band-pass filter had cutoff frequencies at 0.1 and 250 Hz. The filtered EEG signal was sampled at 5 kHz, digitized to 16-bit, and transferred to the recording computer via a fiber-optic cable. The EEG recording system was synchronized with the scanner’s internal clock, which, along with the high sampling rate, was essential to ensure the successful removal of the gradient artifacts.

### FMRI Data Acquisition

Functional MRI images were acquired on a 3-Tesla Philips Achieva whole-body MRI system (Philips Medical Systems, the Netherlands) using a T2*-weighted echoplanar imaging (EPI) sequence (echo time (TE) = 30 ms; repetition time (TR) = 1980 ms; flip angle = 80°). Two hundred and twelve (212) volumes of functional resting state images were acquired, with each whole-brain volume consisting of 36 axial slices aligned with the anterior and posterior commissures (field of view: 224 mm; matrix size: 64 × 64; slice thickness: 3.50 mm; voxel size: 3.5 mm × 3.5 mm × 3.5 mm). A T1-weighted high resolution structural image was also obtained from each subject.

### EEG Preprocessing

There were two major sources of MRI-related artifacts in EEG recorded simultaneously with fMRI: the gradient artifacts and the cardioballistic artifacts. The gradient artifacts were removed by subtracting an average artifact template from the data set as implemented in Brain Vision Analyzer 2.0 (Brain Products GmbH, Germany). The artifact template was constructed by using a sliding-window approach which involved averaging the EEG signal across the nearest 41 consecutive volumes. The cardioballistic artifacts were also removed by an average artifact subtraction method (Allen et al., 1998). In this method, the R peaks were first detected in the ECG recordings by the algorithm in Brain Vision Analyzer, and then visually inspected to ensure accuracy. The appropriately detected R peaks were utilized to construct a delayed average artifact template over 21 consecutive heartbeat events. The cardioballistic artifacts were then removed by subtracting the average artifact templates from the EEG data. After these two steps, the EEG data were band-pass filtered between 0.5 and 50 Hz, down-sampled to 250 Hz, re-referenced to the average reference (Nunez et al., 1997), and exported to EEGLAB (Delorme and Makeig, 2004) for analysis.

### FMRI Preprocessing

Functional MRI data was preprocessed in SPM5^1^. The first 5 scans of the resting state session were discarded in order to eliminate the transient effects. The preprocessing steps included motion correction, slice timing correction, co-registration, normalization to the Montreal Neurological Institute (MNI) template, re-sampling of the functional images into a voxel size of 3 mm × 3 mm × 3 mm (Friston et al., 1995), and spatial smoothing by a Gaussian kernel with 7 mm FWHM (Full Width at Half Maximum). Global scaling was applied to remove the global signal from the BOLD time series (Desjardins et al., 2001). The BOLD time series was then band-pass filtered between 0.01 and 0.1 Hz.

### SOBI ICA Estimation of Mu Components

**FIGURE 1:**
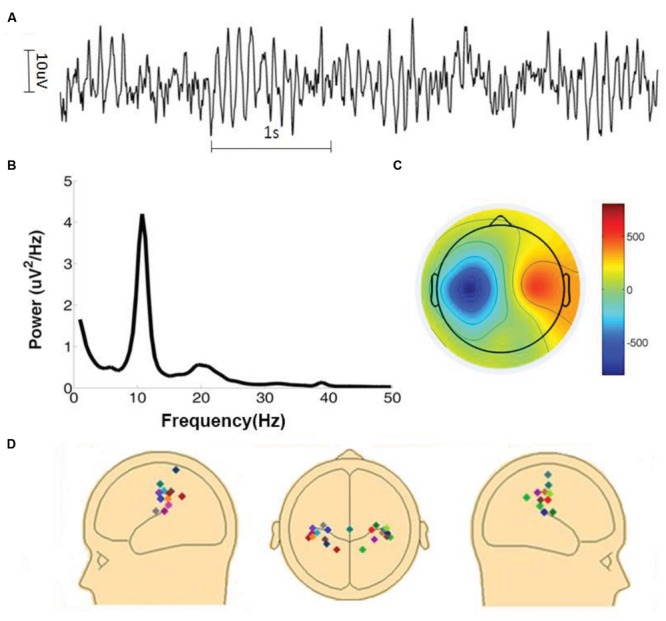
**Identification of mu components. (A)** Time series of a typical mu component. **(B)** Power spectrum of the mu component shown in **(A)**. **(C)** Topographic map of the mu component shown in **(A)**. Color bar on the right side represents the inversed weights of ICA components. **(D)** Dipole sources of all 22 mu components.

The amplitude of mu oscillations is typically much smaller than that of the more dominant posterior alpha oscillations (Benbadis, 2015). Because the two rhythms share similar frequency characteristics, sensorimotor mu activity estimated at the sensor level is likely to be contaminated by visual alpha activity due to volume conduction. To overcome this problem, artifacts-corrected EEG data was decomposed into independent components by the Second Order Blind Identification (SOBI) method (Belouchrani et al., 1997; Tang et al., 2005a,b; Moore et al., 2012) implemented in EEGLAB. An ICA component was selected for further examination if (1) the time series had a power spectrum with a well-defined peak within the 8–12 Hz range, (2) the spatial topographic map suggested a sensorimotor origin, and (3) the time series was not contaminated by large movement artifacts for the entire 7 min of recording and analysis. See **Figures 1A–C** for an example. To further confirm the component’s sensorimotor origin its dipole source was obtained using the DIPFIT function implemented in EEGLAB. The locations of the fitted dipoles, with one dipole for each component, were subject to visual inspection. A total of 22 independent mu components were selected from 15 subjects. For the subjects (*n* = 7) who contributed two components, we tested the dependence of the two components, and found that the correlation between the two components was small (mean *R*^2^ = 0.06). In **Figure 1D** the dipole sources of the 22 components (11 in the left hemisphere and 11 in the right hemisphere) were displayed. The sensorimotor origin of these dipoles were apparent. For the remaining 21 subjects whose ICA components did not meet the above stringent inclusion criteria their data were not considered further.

### Estimation of Mu Power Time Series

For each of the 22 components the mu power time series was estimated by the following steps. First, the EEG signal was segmented into 500 ms non-overlapping epochs. Second, the EEG signal within each epoch was zero–padded to 4 times its original length (500 points after padding) to enhance spectral resolution from 2 to 0.5 Hz. Third, the EEG power spectrum for each epoch was calculated using a non-parametric multitaper approach with 3 tapers (Mitra and Pesaran, 1999), and the mu power of each epoch was estimated by averaging the power spectrum between 8 and 12 Hz. Fourth, for epochs with abnormal log power values (>3 SD), ∼3% of the data, their power was replaced by the average power of the epochs on either sides of them. Fifth, the mu power time series so-obtained was convolved with the canonical HRF. Finally, the HRF-convolved mu power time series was down-sampled to the same sampling frequency as the BOLD signal for correlation analysis.

### Correlation between Mu Power and BOLD Activity

Zero-lag cross correlation between HRF-convolved mu power time series and BOLD time series from all voxels was computed for each mu component and Fisher transformed (Fox et al., 2005; Mantini et al., 2007; Mo et al., 2013). Brain regions showing significant mu-BOLD correlation at the group level (*p* < 0.05, FDR) were identified by a voxel-wise one-sample *t*-test.

### Correlation between Beta and Delta Power and BOLD Activity

To ensure that the mu-BOLD correlation is not due to non-physiological factors such as motion and noise, HRF-convolved beta (18 to 30 Hz) power time series and HRF-convolved delta (1 to 4 Hz) power time series were similarly estimated from the same 22 components, and correlated with the BOLD. Brain regions showing significant beta-BOLD and delta-BOLD correlation at the group level (*p* < 0.05, FDR) were identified by a voxel-wise one-sample *t*-test.

## Results

The time series from a typical mu component is shown in **Figure 1A** where rhythmic activity is clearly seen. In **Figure 1B**, the power spectrum of the component time series confirms the presence of ∼10 Hz oscillation. The sensorimotor origin of this component is suggested by the component’s topographic map in **Figure 1C**. To further verify the origin of a mu component, its topographic map was subject to a source localization procedure. The dipole sources of the 22 components analyzed here were plotted in three different views in **Figure 1D**. These dipole sources were seen to be located in the vicinity of the central sulcus.

**FIGURE 2:**
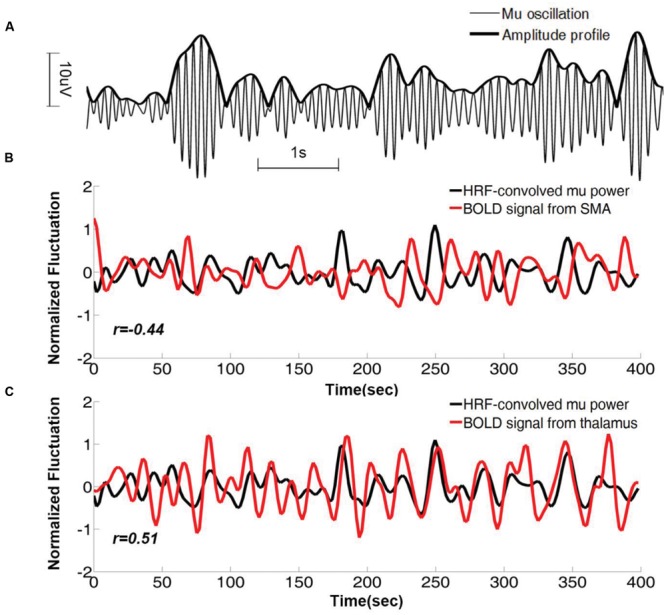
**Mu time series and its correlation with BOLD from a representative subject. (A)** Mu oscillations and its amplitude profile. **(B)** HRF-convolved mu power time series plotted together with the simultaneously acquired BOLD time series from right SMA. Correlation coefficient between the two time series is *r* = -0.43 (*p* < 0.01). **(C)** The same HRF-convolved mu power time series plotted together with the simultaneously acquired BOLD time series from right thalamus. Correlation coefficient *r* = 0.51 (*p* < 0.01). SMA: supplementary motor cortex.

As can be seen in **Figure 1A**, the power (amplitude) of the time series fluctuates over time, and this fluctuation can be used to discover the brain regions whose activities co-vary with it. For each mu component, its mu power time series, which was used to track the fluctuation of the mu amplitude profile (**Figure 2A**), was estimated using a short-time Fourier approach. The HRF-convolved mu power time series, plotted together with BOLD signals from two different brain regions (**Figures 2B,C**) reveal that mu power was negatively correlated with BOLD from SMA but positively correlated with BOLD from thalamus.

**FIGURE 3:**
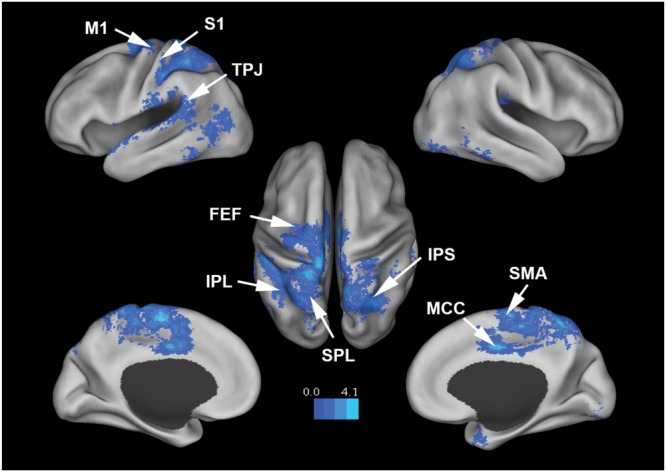
**Brain regions whose BOLD signals are negatively correlated with EEG mu power (*p* < 0.05, FDR corrected).** *T*-values were color-coded. SMA, supplementary motor cortex; MCC, middle cingulate cortex; FEF, frontal eye field; SPL, superior parietal lobule; IPS, intraparietal sulcus; IPL, inferior parietal lobule; M1, primary motor cortex; S1, primary somatosensory cortex; TPJ, temporoparietal junction.

**Figure 3** shows that, at the group level, mu power was negatively correlated with BOLD in a number of brain regions, including (1) SMA, pre- and post-central gyrus, which are major areas in the sensorimotor system, (2) FEF, IPS, and superior parietal lobule (SPL), which are major areas in the attention control system, and (3) IPL, IFG, pMTG, and STG, which are areas variously associated with the mirror neuron system and theory of mind. **Table 1** summarizes the regions, in Automated Anatomical Labeling (AAL) nomenclature, that are negatively correlated with mu power (coordinates of most correlated voxels are listed). The putative membership of each region in major functional brain systems is also given.

**Table 1:** Brain regions (in AAL nomenclature) whose BOLD signals are negatively correlated with EEG mu power (*p* < 0.05, FDR corrected).

Anatomical Regions	MNI Coordinates			*T* values
**Sensorimotor and Motor Control Regions**		
Left supplementary motor area	-3	-3	54	7.88
Right supplementary motor area	6	-12	72	4.38
Left postcentral gyrus	-18	-40	72	4.88
Right postcentral gyrus	33	-40	70	3.98
Left precentral gyrus	-30	-15	66	4.51
Left paracentral lobule	-3	-27	78	6.47
**Attention Control Regions**		
Left superior parietal lobule	-36	-51	60	6.49
Right superior parietal lobule	21	-56	64	4.07
Left superior frontal gyrus	-18	-9	72	5.14
Left precuneus	-11	-42	77	5.53
Right precuneus	6	-54	72	6.71
**Mirror Neuron and Theory of Mind Regions**		
Left inferior parietal lobule	-45	-33	42	4.66
Left superior temporal gyrus	-60	-27	9	5.96
Right superior temporal gyrus	45	-30	12	4.56
Left middle cingulate cortex	-1	-3	48	6.64
Left superior temporal pole	-54	12	-12	4.99
Left middle temporal gyrus	-60	-51	12	4.65
Right parahippocampal	21	9	-30	4.49
**Other Regions**		
Left middle occipital gyrus	-48	-72	12	4.85
Left inferior temporal gyrus	-60	-54	-18	4.68
Right inferior temporal gyrus	51	-39	-24	4.52

*FEF, IPS, and TPJ, are contained in superior frontal gyrus, superior parietal lobule, superior temporal gyrus clusters, respectively.*

**FIGURE 4:**
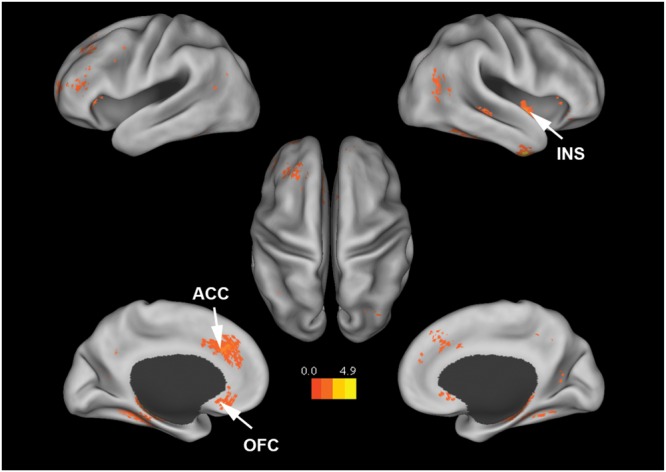
**Brain regions whose BOLD signals are positively correlated with EEG mu power (*p* < 0.05, FDR corrected).** *T*-values were color-coded. INS, insula; ACC, anterior cingulate cortex; OFC, orbital frontal cortex.

**Figure 4** shows that, at the group level, mu power was positively correlated with BOLD signals from anterior cingulate cortex (ACC), insula, and orbital frontal cortex. **Table 2** lists these regions and additional cortical and subcortical areas including thalamus, putamen, and hippocampus whose BOLD was also positively correlated with mu power. Many of these structures lie within the salience network (Seeley et al., 2007), which is thought to play a crucial role in the detection of salient stimuli such as error, threat, faces of loved ones, and social rejection (Eisenberger et al., 2003; Bartels and Zeki, 2004; Holroyd et al., 2004). Other subcortical structures in the salience network are variously associated with such diverse functions as reward, emotion, and homeostasis.

**Table 2:** Brain regions whose BOLD signals are significantly positively correlated with EEG mu power (*p* < 0.05, FDR corrected).

Anatomical Regions	MNI Coordinates			*T* values
**Salience Network Regions**		
Left insula	-33	27	0	3.56
Right insula	39	0	-6	4.36
Left anterior cingulate cortex	-11	27	33	4.29
Right medial orbital frontal cortex	3	27	-9	3.39
Right putamen	33	0	0	5.00
Left caudate	-12	15	12	3.73
Right caudate	15	3	24	3.58
Left hippocampus	-27	-9	-15	5.76
Right hippocampus	33	-30	-3	5.16
Left thalamus	-10	-9	0	4.12
Right thalamus	21	-17	0	3.85
**Other Regions**		
Left fusiform	-33	-33	-15	6.29
Right inferior temporal gyrus	42	3	-45	4.91
Left middle frontal gyrus	-30	39	48	4.59
Right medial prefrontal cortex	9	39	45	4.18
Left cuneus	-15	-54	21	4.02
Right inferior frontal gyrus	39	39	-9	3.63
Left rectus	-3	27	-15	3.40
Right superior frontal gyrus	15	48	24	3.15

**FIGURE 5:**
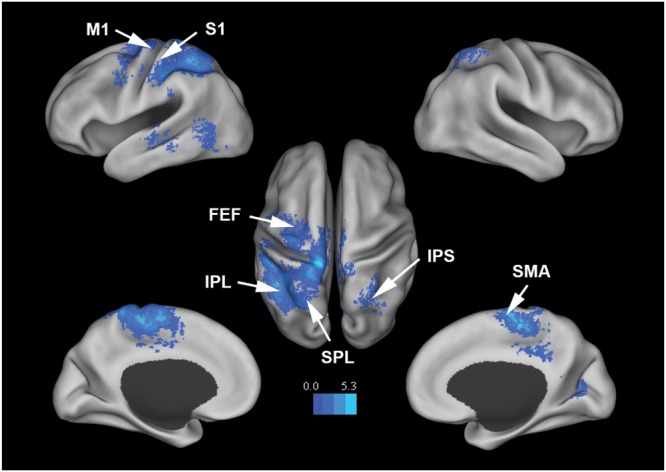
**Brain regions whose BOLD signals are negatively correlated with EEG beta power from the 22 mu components (*p* < 0.05, FDR corrected).** *T*-values were color-coded.

In additional analysis, HRF-convolved beta (18 to 30 Hz) power time series and HRF-convolved delta (1 to 4 Hz) power time series were similarly estimated from the same 22 components, and correlated with the BOLD. As shown in **Figure 5**; **Table 3**, beta power was negatively correlated with BOLD from SMA, M1, FEF, IPS, and other motor and attention control regions. No areas were positively correlated with beta power under *p* < 0.05, FDR. Delta (1∼4 Hz) power was not correlated with any brain regions under *p* < 0.05, FDR.

**Table 3:** Brain regions (in AAL nomenclature) whose BOLD signals are negatively correlated with EEG beta power (*p* < 0.05, FDR corrected).

Anatomical Regions	MNI Coordinates			*T* values
Left supplementary motor area	-3	-3	54	4.06
Left postcentral gyrus	-30	-33	63	4.12
Right postcentral gyrus	9	-42	78	3.96
Left precentral gyrus	-30	-12	66	7.13
Left paracentral lobule	0	-30	57	6.72
Right superior parietal lobule	36	-51	63	4.67
Left precuneus	-9	-45	72	6.18
Right precuneous	6	-40	42	4.49
Left superior temporal gyrus	-45	-39	15	4.24
Left middle temporal gyrus	-54	-66	6	4.03

*The regions included in this table are a subset of the regions reported in **Table 1**.*

## Discussion

Event-related modulation of the mu rhythm has been variously interpreted as reflecting the activation of the motor system, the attention control system, or the mirror neuron system (McFarland et al., 2000; Oberman et al., 2005; Pineda and Hecht, 2009; Anderson and Ding, 2011). We examined the physiological foundation of such interpretations by recording simultaneous EEG-fMRI during resting-state. Correlating mu power fluctuations with BOLD, our results showed that the mu rhythm was negatively correlated with BOLD in (1) SMA and pericentral cortex of the sensorimotor system, (2) FEF, IPS, and SPL of the attention control system, (3) STG, TPJ and TP of theory of mind related regions, as well as (4) IFG and IPL of the putative mirror neuron system. In addition, mu power was also found to be positively correlated with BOLD in ACC, orbital frontal cortex, insula, thalamus, and putamen, areas of the salience network (Murta et al., 2015).

### Mu Rhythm and Motor Control

Observation of event-related desynchronization (ERD) of mu with motor preparation and execution dates back to the 1950s (Chatrian et al., 1959). Pfurtscheller and Neuper (1997) later reported that motor imagery could also lead to the ERD or suppression of mu rhythm. Recent neural stimulation studies provide additional physiological insight into mu modulation. After anodal transcranial direct current stimulation (tDCS) of the primary motor cortex, mu ERD was found to be significantly enhanced during motor imagery, whereas cathodal stimulation of the same area reduced mu ERD (Matsumoto et al., 2010), suggesting that the power of mu is inversely related to the excitability of the sensorimotor cortex. These findings are in accord with our result that BOLD in sensorimotor cortex and higher order motor control areas such as SMA is negatively correlated with mu power.

### Mu Rhythm and Attention Control

Mu rhythm is reported to be modulated by spatial somatosensory attention in recent studies (Jones et al., 2010; Anderson and Ding, 2011; Haegens et al., 2011). Specifically, mu power is decreased with increased attention, signifying increased excitability of the somatosensory cortex to enhance the processing of attended information. Our finding that mu power is negatively correlated with BOLD from attention control areas including SPL, IPS, and FEF is consistent with this observation. In addition, given the prominent role played by SPL, IPS, and FEF in visual attention control, our result lends support to the proposal that there exists a “supramodal” attention control network, encompassing frontoparietal regions SPL, IPS, and FEF, that mediates goal-directed attention in multiple sensory modalities (Macaluso and Driver, 2001; Eimer and Van Velzen, 2002).

### Mu Rhythm and the Mirror Neuron System

Mirror neurons are a class of neurons that modulate their activity both when an individual executes a specific motor act and when they observe the same or similar act performed by another individual (Kilner and Lemon, 2013). Mirror neurons are thought to play an important role in transforming sensory information into motor acts (Rizzolatti and Fogassi, 2014). Intriguingly, observing actions can also induce the suppression of mu, leading to the notion that mu suppression could also reflect the activation of the mirror neuron system (Pineda, 2005; Oberman et al., 2005). This notion has found clinical applications in the study of the autism spectrum disorder (Oberman et al., 2005). While the exact locations of the mirror neuron system in humans are debated (Le Bel et al., 2009), regions such as premotor cortex, IPL, pMTG and even somatosensory cortex are hypothesized to be the components of the mirror neuron system in humans (Gazzola et al., 2006; Pineda, 2008). Our finding that BOLD from these areas is negatively correlated with mu power supports the notion that mu rhythm can index the activity of the mirror neuron system.

### Mu Rhythm and Theory of Mind

Comprehending the mental state of others through expressed experience and behavior is an area of social cognition called theory of mind. Theory of mind may engage the mirror neuron system as well as other processes (Frith, 2001; Uddin et al., 2007; Pineda and Hecht, 2009). Functional imaging studies have found theory of mind related activities in TP, TPJ, and STS (Gallagher and Frith, 2003; Saxe and Kanwisher, 2003). Our data showing that the BOLD activity in these regions is negatively correlated with mu power suggests for the first time that theory of mind related regions are associated with mu rhythm. Furthermore, the middle cingulate cortex (MCC), known to play a role in mentalizing (Chiu et al., 2008; Lombardo et al., 2010; Lombardo and Baron-Cohen, 2011), is one of the regions strongly negatively correlated with mu rhythm in our data.

### Mu Rhythm and the Salience Network

Blood-oxygen-level-dependent from areas of the salience network including ACC, orbital frontal cortex, insula, putamen, and thalamus is positively correlated with mu power. It has been proposed that the salience network serves to detect salient events, both external and internal (Palaniyappan and Liddle, 2012), and facilitate access to attention, working memory and motor resources (Menon and Uddin, 2010). Our results appear to suggest that during initial stages of salience detection and perceptual decision-making, when the salience network is activated (Lamichhane and Dhamala, 2015), the motor system is immobilized. Recent behavioral and stimulation studies have identified a freezing-type of response when human subjects are exposed to salient input such as sexually explicit stimuli (Mouras et al., 2015), emotionally engaging pictures (Azevedo et al., 2005), or even fearful body language (Borgomaneri et al., 2015). Using TMS, Borgomaneri et al. (2015) found an early reduction of excitability in the motor system when subjects were exposed to fearful body language. More directly, in a resting-state fMRI study, anterior insula, a core component of the salience network, is found to be negatively correlated with the somatosensory cortex (Cauda et al., 2011), suggesting that BOLD from the salience network may be positively correlated with mu power. Although a direct link between the salience network activation and mu rhythm has not been suggested before, past work demonstrates the plausibility of such a link.

### Mu Rhythm and Thalamocortical Activity

It is a long-held notion that brain oscillations involve recurrent thalamocortical activity (Steriade et al., 1990; Llinás et al., 2005). While the cortex provides feedback to the thalamus through corticothalamic projections to modulate thalamic responses, or to synchronize oscillations on a large scale (Golshani et al., 2001), the thalamus often initiates its rhythmic activity before, and terminates after, the cortex, with the reverse pattern never occurring (Bouyer et al., 1982). In addition, thalamic mu oscillatory activity was found to lead cortical mu activity by several hundred milliseconds (Buzsaki, 1991), whereas cortical mu activity was not observed in the absence of thalamic mu activity (Semba et al., 1980). Our result that the activity of the thalamus is positively coupled with mu power agrees with the notion that sensorimotor EEG rhythm has an origin in the thalamus (Andersen and Andersson, 1968). Examining the temporal relation between the cortex and the thalamus within mu rhythm range, however, is beyond the temporal resolution of BOLD.

### Comparison with Visual Alpha Rhythm

Alpha-BOLD coupling has been extensively studied using the simultaneous EEG-fMRI method (Goldman et al., 2002; Gonçalves et al., 2006; Mo et al., 2013). One feature common to the alpha-BOLD correlation map and the mu-BOLD correlation map is that both contain the areas in dorsal attention network (DAN) such as FEF and IPS. The fact that BOLD from these regions is negatively correlated with both alpha and mu suggests the importance of alpha and mu as sensory indicators of attentional influences as well as provides support to the supramodal control of attention theory. Another common feature is that both mu and alpha are positively correlated with the thalamic BOLD signal (Goldman et al., 2002; Martínez-Montes et al., 2004; de Munck et al., 2007; Liu et al., 2012). This is in agreement with the idea that the thalamus plays a central role in the generation and modulation of cortical rhythms (da Silva et al., 1973). One obvious difference between the two maps is that the alpha-BOLD correlation map is associated with visual cortex whereas the mu-BOLD correlation map is associated with sensorimotor cortex. In addition, mu appears to be more involved in social cognition and perception by the virtue of its negative coupling with the mirror neuron system and theory of mind areas; alpha is not linked to these systems. The difference also exists in the positive correlations as alpha is positively correlated with the default mode network (Mo et al., 2013) whereas mu is shown here to be positively correlated with the salience network.

### Methodological Considerations

First, the foregoing demonstrates that visual alpha and sensorimotor mu are different neural constructs. The small correlation of their temporal fluctuations is consistent with this idea (*R*^2^= 0.037 ± 0.001). These suggest that deriving a single 10 Hz power from the entire brain is not advisable because doing so will mix the sources that generate and modulate the two different brain rhythms. Second, mu estimated on the scalp is likely to be contaminated by visual alpha, and this could be the reason why previous resting-state EEG-fMRI studies did not find any difference in EEG-BOLD correlation maps between 10 Hz EEG activity derived from occipital channels (O1/O2) and from central-parietal channels (C3/C4) (Laufs et al., 2003). Subsequent studies using scalp-estimated-mu found coupling between mu and sensorimotor activity but not between mu and activities from other higher order cognitive brain regions (Ritter et al., 2009; Braadbaart et al., 2013). We overcame this problem here by applying ICA to isolate the mu components. Third, it has been demonstrated that motion artifacts could impact task EEG data even after stringent cleaning (Jansen et al., 2012). Although resting state recording generally contains less motion artifacts than task state recording, we nevertheless tested whether our findings were significantly impacted by such artifacts. Beta-band and delta-band power were derived from the mu components and correlated with BOLD. Beta-BOLD and mu-BOLD negative correlation maps both contained motor control areas and attention control areas, as expected (McFarland et al., 2000; de Munck et al., 2009; Meirovitch et al., 2015), but the delta-BOLD correlations were not significant, and there is no significant positive beta-BOLD correlations. These analyses suggest that the reported findings on mu-BOLD relationships are unlikely to be dominated by a global factor such as motion. Fourth, classification of brain regions into different functional brain systems (**Tables 1** and **2**) can only be considered approximate. Some regions are known to have multiple functions. For example, TPJ is a theory of mind region, but it is also part of the ventral attention network.

### Comparison with Resting-state Networks

It is instructive to compare regions in **Tables 1** and **2** with frequently reported resting-state networks. For example, the coordinates of left SPL, right SPL, and left superior frontal gyrus, which were reported as attention control regions in **Table 1**, are contained within the DAN (Duan et al., 2012; Lee et al., 2012), whereas the coordinates of left insula, right insula, and left anterior cingulate, which were reported as salience network regions in **Table 2**, are contained within the salience network (van den Heuvel et al., 2008; Orliac et al., 2013). This observation suggests an alternative method to uncover the brain networks underlying the generation and modulation of rhythmic brain activity. In future studies one may attempt to first use ICA to identify resting-state networks and then correlate the rhythmic brain activity with BOLD from these networks to assess their relationship.

## Author Contributions

SY analyzed the data, interpreted the result, and wrote the paper with MD, YL collected the data and made very useful suggestions to the project. MD designed the study, developed the methodology, and offered interpretation to the results.

## Conflict of Interest Statement

The authors declare that the research was conducted in the absence of any commercial or financial relationships that could be construed as a potential conflict of interest.

## Footnotes

1 http://www.fil.ion.ucl.ac.uk/spm/
